# Utilizing Intraoperative Navigated 3D Color Doppler Ultrasound in Glioma Surgery

**DOI:** 10.3389/fonc.2021.656020

**Published:** 2021-08-18

**Authors:** Benjamin Saß, Mirza Pojskic, Darko Zivkovic, Barbara Carl, Christopher Nimsky, Miriam H. A. Bopp

**Affiliations:** ^1^Department of Neurosurgery, University of Marburg, Marburg, Germany; ^2^Department of Neurosurgery, Helios Dr. Horst Schmidt Kliniken, Wiesbaden, Germany; ^3^Center for Mind, Brain and Behavior (CMBB), Marburg, Germany

**Keywords:** intraoperative ultras, color Doppler ultrasound, intraoperative imaging, brain shift, glioma

## Abstract

**Background:**

In glioma surgery, the patient’s outcome is dramatically influenced by the extent of resection and residual tumor volume. To facilitate safe resection, neuronavigational systems are routinely used. However, due to brain shift, accuracy decreases with the course of the surgery. Intraoperative ultrasound has proved to provide excellent live imaging, which may be integrated into the navigational procedure. Here we describe the visualization of vascular landmarks and their shift during tumor resection using intraoperative navigated 3D color Doppler ultrasound (3D iUS color Doppler).

**Methods:**

Six patients suffering from glial tumors located in the temporal lobe were included in this study. Intraoperative computed tomography was used for registration. Datasets of 3D iUS color Doppler were generated before dural opening and after tumor resection, and the vascular tree was segmented manually. In each dataset, one to four landmarks were identified, compared to the preoperative MRI, and the Euclidean distance was calculated.

**Results:**

Pre-resectional mean Euclidean distance of the marked points was 4.1 ± 1.3 mm (mean ± SD), ranging from 2.6 to 6.0 mm. Post-resectional mean Euclidean distance was 4.7. ± 1.0 mm, ranging from 2.9 to 6.0 mm.

**Conclusion:**

3D iUS color Doppler allows estimation of brain shift intraoperatively, thus increasing patient safety. Future implementation of the reconstructed vessel tree into the navigational setup might allow navigational updating with further consecutive increasement of accuracy.

## Introduction

Gliomas are the most common primary brain tumors representing 27% of all brain and central nervous system (CNS) tumors and 80% of malignant brain tumors in the United States (US) population (1). The broad category of gliomas encompasses tumors of astrocytic, oligodendrocytic, or ependymal origin and is classified by the World Health Organization (WHO) into four grades, depending on histological and molecular characteristics (2). Grade I tumors, typically seen in children, are potentially curable when resected, whereas low-grade gliomas (WHO grade II), which are mostly seen in young adults, progress eventually to high-grade gliomas (3). The majority of gliomas (55.1%) are glioblastomas WHO grade IV, which occur with an incidence of 3.4 per 100,000 (1). The main cornerstones of glioma therapy include surgery for histological diagnosis and tumor removal, radiotherapy, and pharmacotherapy (4). There is an ongoing debate on the appropriate resection strategy, mainly driven by the demonstration of glioma cells within regions appearing to be normal brain tissue on cerebral magnetic resonance tomography (MRI) (5) and computed tomography (CT) (6, 7) and even in histologically normal brain regions (8). Several studies have proved the extent of resection (EOR) and the residual tumor volume in glioma surgery to be important factors influencing the patient’s outcome as measured in progression-free survival and overall survival (9–12). Thus, it is common practice to resect as much tumor as possible while preserving neurological functions (13).

Prerequisite for this is the localization of pathological tissue as well as eloquent brain areas during the neurosurgical procedure, which can be realized using neuronavigational systems. These systems commonly utilize preoperative imaging, to which the patient is registered (14). Intraoperative imaging modalities such as computed tomography (iCT) (15–17), magnetic resonance tomography (iMRI) (18–20), and ultrasound (iUS) (21–23) can be integrated into these systems, improving safety and accuracy. Besides the possibility of instant resection control, intraoperative imaging can help the neurosurgeon to deal with brain shift, a well-described phenomenon, which is mostly due to brain swelling, loss of cerebrospinal fluid, tumor reduction, brain retraction, and influences of gravity after craniotomy and dural opening (24, 25). First efforts to estimate the extent of brain deformation date back to the 1980s (26). Since then various attempts have been made to approach this issue including optical scanning (27) and navigated pointer based surface displacement measurements (28, 29), a stereotactic system with integrated operative microscope and video analysis (24), iMRI (30, 31), and iUS (32–34). Brain shift has been shown to occur during the whole operative procedure, which can be partially addressed by serial MRI acquisitions as demonstrated by Nabavi et al. (35). The main limitations of iMRI are its restricted availability, structural requirements, time consumption, and high costs (36, 37). None of these drawbacks apply for iUS, which can be performed without significant interruption of the surgical procedure, is nowadays widely available, straightforward in use, and cost-effective (38). Modern ultrasound systems can be fully integrated into neuronavigational setups (39, 40) and are able to provide the neurosurgeon with information about resection extent in glioma surgery (40, 41) and brain deformation (39).

First descriptions of brain shift measurements utilizing iUS were published in the late 1990s, when specific, easily identifiable structures like the ventricles were marked in preoperative and intraoperative imaging to evaluate brain shifting (32–34). In 2003 Keles et al. analyzed pre- and postresectional navigated iUS for brain shift correction and determination of resection extent (42). By that time iUS image quality and integration into navigational setups were rather poor, but improved successively the following years (39, 40, 43). In 2010 Ohue et al. described a new US-linked navigation system with improved imaging quality, which they used to quantify brain deformation at different anatomical (ventricles, meninges, sulci) or pathological structures (tumor boundaries) before and after dural opening and after tumor resection (22). These structures were identified in intraoperative brightness modulation (B-mode) ultrasound, whereas vessels can be visualized better in color or power Doppler mode. Already in 2001, Slomka et al. described a voxel-based registration of 3D Doppler ultrasound and preoperative MRI datasets using an iterative algorithm searching for the best geometric match in six cases (44). Rasmussen et al. examined postoperative automatic fusion of magnetic resonance angiogram (MRA) and intraoperative 3D Doppler ultrasound (i3D US Doppler) in five cases and found satisfactory results in terms of accuracy and time expenditure (45). In 2007 Reinertsen et al. intensified research in this respect and validated retrospectively an algorithm in five patients that used vascular centerlines extracted from both modalities to eventually achieve non-linear registration (46). The aforementioned investigations were feasibility studies outside the operating theater, and it took until 2014 to implement intraoperative use, when Reinertsen et al. reported an ultrasound-based registration method to correct for brain shift running during surgery. They performed semi-automatic registration based on MRA and i3D US Doppler datasets, in which the vascular tree was segmented, in seven cases (three vascular and four tumor procedures) and reported their method to be fully integrated into the neuronavigational system and ready to use (37). I3D US Doppler can also be sufficiently co-registered to other vascular imaging modalities, such as 3D digital subtraction angiography (DSA), as shown in 37 vascular cases by Podlesek et al., who utilized a curved linear array transducer capable of generating 3D volumes, which is typically used for endocavitary examinations in obstetrical, gynecological, and urological applications. They described iUS to be a valuable adjunct to established intraoperative vascular imaging modalities like indocyanine green angiography (ICG), although they did not integrate iUS into the navigational setup (47). Mohammadi et al. proposed and tested in a phantom and animal model a new approach for brain shift estimation utilizing a combination of surface imaging (stereo vision) and iUS Doppler, which were both registered to preoperative MRI respectively MRA datasets (48, 49). However, this approach has so far not entered clinical practice.

In our institution, i3D US datasets are routinely acquired during brain tumor resections, adding intraoperative live imaging to our multimodal neuronavigational setup. We have shown for brain metastasis that i3D US clearly delineates tumor boundaries and thereby allows pathologically based estimation of brain shift (50). Whilst tumor contours clearly identifiable with i3D US allow object analyzation and consecutive brain shift estimation in metastasis surgery, this becomes more challenging in glioma surgery, where tumor boundaries are often not well defined in iUS. In the presented prospective work, we focused on brain shift estimation in glioma surgery utilizing preoperative MRI (preMRI) and i3D US Doppler datasets for the visualization of vascular structures. We designed this work as a proof-of-concept study to examine how the analysis of the vascular shift can be implemented in the preexisting navigational setup with as little workaround as possible to make it easily applicable for intraoperative use.

## Materials and Methods

To allow good visualization of vessels in i3D US, we only included patients suffering from gliomas and one case of a dysembroplastic neuroepithelial tumor (DNET), which were operated utilizing a temporal craniotomy. For more details see **Table 1**. Informed consent was obtained from all patients. Ethics approval was granted for prospective archiving clinical and technical data applying intraoperative imaging and navigation (study no. 99/18).

**Table 1:** Patient characteristics and tumor volume.

Case	Age [Years]	Diagnosis	Tumor volume [cm^3^]
1	62.4	GBM	47.8
2	37.0	DA	71.5
3	40.8	GBM	39.9
4	71.4	DA*	67.5
5	35.2	DNET	20.4
6	55.6	GBM	41.3

GBM, glioblastoma; DA, diffuse astrocytoma; DA*, DA with molecular features of a glioblastoma WHO grade IV; DNET, dysembroplastic neuroepithelial tumor.

All patients underwent MRI imaging within a few days before surgery, typically including contrast-enhanced imaging and time-of-flight (ToF) sequences for vascular imaging. Preoperative imaging was transferred to the navigational system (Brainlab, Munich, Germany), consisting of a ceiling-mounted double monitor (Curve, Brainlab, Munich, Germany), a wall-mounted double display (Buzz, Brainlab, Munich, Germany), and navigational software.

All procedures were conducted under general anesthesia, and all patients received 40 mg of dexamethasone. After narcosis induction, the patients were positioned, the head placed horizontally and fixed to the OR (operating room) table using a radiolucent Doro head clamp with metallic pins. The pins were placed in such a way that in between intraoperative computed tomography (iCT) scanning for registration was possible without significant artifacts. A reference array with four reflective markers was attached to the head clamp. Although not necessary for registration, three fiducial markers were placed on the patient’s head within the scanning range allowing registration accuracy measurements. After a 90° rotation of the OR table to the 32-slice mobile CT scanner (AIRO, Brainlab, Munich), a low-dose registration scan (0.042 mSv) of 62 mm scan length was performed. During the scanning process the navigational camera detected reflected markers permanently attached to the AIRO-scanner and the reference array. The dataset was automatically transferred to the navigational system and fused to the preoperative imaging data to establish patient registration. After rotating the OR table back, the registration accuracy was checked by placing the tip of the navigational pointer in the divot of each of the three fiducial markers, which allowed calculation of the target registration error (TRE) as the Euclidean offset of the pointer tip. The reference array was removed and replaced by a sterile one after surgical skin preparation and sterile draping. Further details on the setup using iCT as an registration device were published before (51).

After team time-out, the skin was incised, and the temporalis muscle dissected. Subsequently, 125 ml of 15% mannitol were administered, and a temporal craniotomy performed. First sets of i3D US and i3D US Doppler data were acquired before dural opening using the ultrasound device bk5000 (bk medical, Herlev, Denmark) with a high resolution, small footprint transducer (N13C5, bk medical, Herlev, Denmark), which has a convex contact surface of 29 × 10 mm, a frequency range of 5–13 MHz, and is fully immersible and sterilizable. The pre-calibrated US probe was equipped with a reference array with three reflective markers. Saline was used as a coupling fluid, and the probe was swept gently over the dural layer for image acquisition. The generated 0.3 mm 2D slices were automatically transferred to the navigational system and transformed to co-registered 3D datasets. During the further surgical procedure, the i3D US and i3D US Doppler datasets were displayed either in an overlay view, side-by-side, or as standalone. After tumor resection, another set of US Doppler images was acquired in the same way in six cases.

For analysis of the vascular displacement, the vascular tree was segmented manually using a threshold-based filtering approach or outlined manually in ToF or T1 contrast-enhanced sequences. Because currently, the software does not allow threshold-based segmentation in i3D US Doppler datasets, in ultrasound images the vascular structures were segmented manually with the smart brush application (Brainlab navigational software, Munich, Germany), which is a computer-assisted outlining tool, allowing accelerated object segmentation. Representative, easily identifiable structures, such as vascular bifurcations, were marked in each dataset. To gain information on the brain deformation before dural opening, the Euclidean distance of the defined landmarks in preoperative MRI (preMRI) and pre-resectional i3D US Doppler was calculated. The brain shift occurring during the operative course was determined using the distance of the corresponding structures in post-resectional i3D US Doppler compared to preMRI.

GraphPad Prism 8.4.3 (GraphPad Software, San Diego, USA) for MacOS was used for statistical analysis. Under the assumption of normally distributed data, a paired t-test was used for further analysis. A p-value <0.05 was considered statistically significant.

## Results and Discussion

Six patients with temporal glial tumors were included in this study. The mean ± standard deviation (SD) patient age was 50.4 ± 14.9 years, ranging from 35.2 to 71.4 years. The mean ± SD tumor volume was 48.07 ± 19.00 cm^3^ (range: 20.4 to 71.5 cm^3^). Histopathological workup revealed glioblastoma in three cases. One case was classified as a diffuse astrocytary glioma with molecular features of a glioblastoma WHO grade IV, and one patient was diagnosed with WHO grade II diffuse astrocytoma. We also included one patient with a temporal DNET. The mean TRE was 0.82 ± 0.11 mm (mean ± SD), depicting an excellent registration accuracy. Patient characteristics and tumor volume are summarized in **Table 1**.

**Figure 1:**
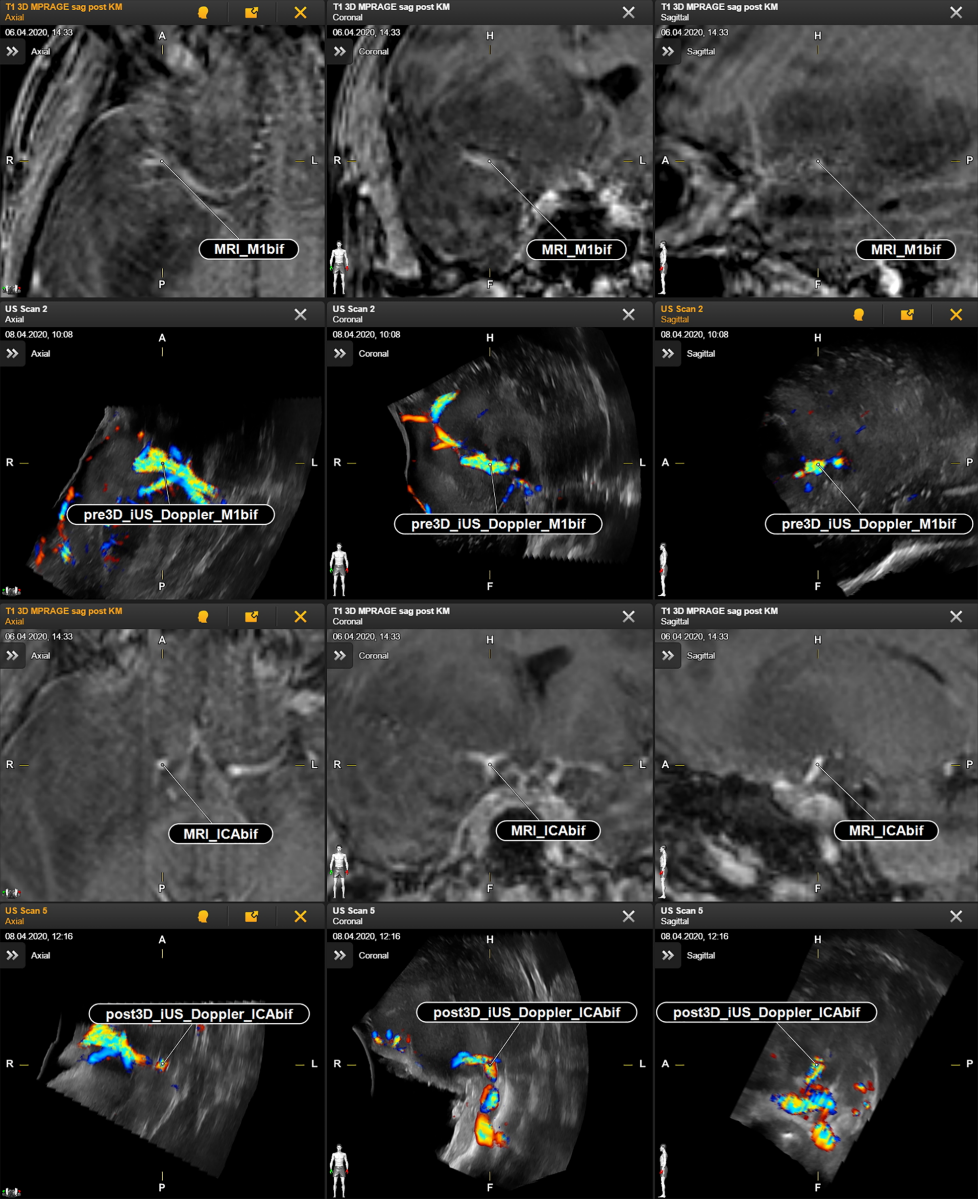
Case no. 2. Identification of vascular anatomic landmarks in MRI and navigated reformatted 3D iUS Doppler. First and second row: preoperative MRI and corresponding pre-resectional 3D iUS Doppler in axial, coronal, and sagittal plane. Third and fourth row: preoperative MRI and corresponding post-resectional 3D iUS Doppler in axial, coronal, and sagittal plane. CAbif, internal carotid artery bifurcation; M1bif, M1bifurcation; pre3D iUS Doppler, pre-resectional 3D iUS Doppler; post3D iUS Doppler, post-resectional 3D iUS Doppler.

**Figure 2:**
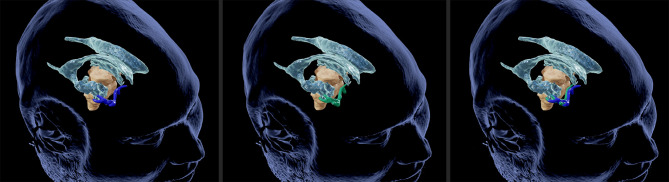
Illustrative case no. 2. 3D view of vascular landmarks segmented in MRI (left) and US (middle). Combined presentation of both segmentations (right) depicts the spatial overlap.

The generation of 3D iUS Doppler image sets was straightforward. Pre-resectional 3D iUS Doppler was performed in all cases before dural opening. In case no. 3, the 3D iUS Doppler image set was corrupted by artifacts, primarily caused by dural thickening, permitting no reliable identification of bifurcations or other prominent vascular structures. In all other cases, one to four vascular anatomic points were identified and the Euclidean distance to the corresponding preMRI data calculated (**Figure 1**). For each case the mean Euclidean distance of the marked points was calculated, ranging from 2.6 to 6.0 mm (mean ± SD = 4.1 ± 1.3 mm). Post-resectionally, 3D iUS Doppler was conducted in all six cases. Here, we found a Euclidean distance of 4.7. ± 1.0 mm (mean ± SD), ranging from 2.9 to 6.0 mm. All measurements are shown in **Table 2**. Interestingly, apart from one measured value in case no. 2, the measured Euclidean distances within each case did not vary much, suggesting that the mean value can give a good indication for the estimation of the local brain deformation. Interestingly, according to our measurements the extent of pre-resectional and post-resectional shift of vascular landmarks is similar (mean of differences: 0.55 mm), without statistically significant differences (p = 0.625; paired t-test), as shown in an illustrative example in **Figure 2**. This is in contrast to the generally accepted assumption that the main shifting occurs after durotomy, as shown by Hill et al., who found a dural displacement of only 1.2 ± 2.0 mm (mean ± SD) after craniotomy, but a brain shift of 4.4 ± 1.9 mm (immediately after dural opening) and of 5.6 ± 1.9 mm (approximately 1 h later), respectively (29). On the other hand, Ohue et al. described a displacement of tumor margins of 3.4 ± 1.9 mm (mean ± SD) after craniotomy but before durotomy, which increased to 5.1 ± 2.7 mm, when the dura mater was opened (22). Similar findings were reported by Lettboer et al., who found the main displacement after craniotomy but reported an additional shift of only 0.2 mm once the dural layer was opened (52). Regarding the pre-durotomy shift, our results are well in line with those of Ohue et al. and Lettboer et al., but one would expect more additional brain deformation after tumor resection as demonstrated, for example, by Roberts et al., who found a mean displacement of 1 cm (24). Yet there is a high variability in the extent of brain shift (25), and particularly deeper located structures are less prone to shifting (25, 53), which could also apply for the vascular structures and thus explain our results. Sastry et al. attributed the observation of a rather small post-durotomy shift to registration errors (38). In an earlier study, we determined the accuracy of the co-registration of the precalibrated ultrasound probe, using a tracked ultrasound phantom containing wires, and found an offset of 1.33 ± 0.33 (mean ± SD) (50). Given this and the here calculated high registration accuracy, we consider the influence of the registrational inaccuracies to be minor on our results. Care has to be taken when interpreting our results of the rather small brain shift of deeper located vessels in this study, as in many surgical situations already a little spatial offset of vascular structures could have devastating effects, if not considered by the surgeon. With that in mind, Šteňo et al. described the visualization of lenticulostriate arteries during the resection of insular low-grade gliomas using navigated 3D power Doppler and found this to be a promising approach to increase safety during the surgical course (54).

**Table 2:** Measurements.

Case	pre3D iUS Doppler *vs.* preMRI			post3D iUS Doppler *vs.* preMRI		
	Vascular Structure	Euclidean Distance [mm]	Mean Euclidean Distance [mm]	Vascular Structure	Euclidean distance [mm]	Mean Euclidean Distance [mm]
1	ICAbif	5.1	6.0	ICAbif	4.9	5.0
	M1bif	6.9		M1bif	5.0	
2	ICAbif	3.8	3.5	ICAbif	2.4	2.9
	M1bif	3.4		M1bif	3.4	
	M2bif	4.9		M2bif	3.0	
	M1cont	1.86		M1cont	2.9	
3	–	–	–	ACIbif	5.2	5.2
				Basilar tip	5.1	
4	ICAbif	4.5	4.3	ICAbif	3.4	4.5
	M1bif	4.0		M1bif	5.5	
5	–		4.0	ICAbif	6.2	6.0
	M1bif	4.0		M1bif	5.7	
6	ICAbif	3.2	2.6	ICAbif	6.2	4.8
	M1bif	2.0		Basilar tip	4.1	
				A2	4.2	

A2, A2 segment of anterior cerebral artery; ICAbif, internal carotid artery bifurcation; M1bif, M1bifurcation segment of the MCA; M1cont, vascular contact of M1 to another vascular branch; M2, M2 segment of MCA; MCA, middle cerebral artery; pre3D iUS Doppler, pre-resectional 3D iUS Doppler; post3D iUS Doppler, post-resectional 3D iUS Doppler.

During surgery the main region of interest with respect to brain shift is the tumor surrounding area, and the actual brain deformation present there might be underestimated by measuring the movement of distant vascular landmarks. In our study, i3D US color Doppler did not depict small peritumoral vessels with such a high resolution that allowed analysis of shifting when compared to preMRI. Regarding this, a reasonable alternative to color Doppler is power Doppler, which does not measure the velocity and direction of the Doppler signal (and thus blood flow) but the power (amplitude) of the signal (55). Power Doppler allows detection of smaller vessels with less blood flow (55, 56), such as peritumoral vascular structures, and is less prone to typical color Doppler limitations and artifacts, like angle dependency or aliasing. Color Doppler is angle dependent, because it depicts the velocity along the ultrasound beam direction and not the true blood flow velocity, and thus, it is not able to detect flow perpendicular to the ultrasound beam. Aliasing occurs when the pulse rate limitation of the ultrasound transducer is exceeded or with inadequate velocity scale settings, resulting in incorrect pixel values. Neither applies for power Doppler, because it is not based on velocity measurements (55, 57). Additionally, power Doppler has less noise artifacts, which can be influenced by Doppler gain settings (38, 57). On the other hand, the power Doppler signal has a tendency to extend beyond the borders of vessels, which consequently appear thicker than they actually are, and the visualization of too many surgically not important vessels might result in rather confusing imaging (58).

Recently, contrast-enhanced ultrasound (CEUS) has been introduced as a real-time imaging in neurosurgical procedures, facilitating identification of pathological tissue and tumor blood supply (59). Ilunga-Mbuyamba et al. acquired intraoperative CEUS and compared it to preoperative MRI data, using an algorithm for image registration. They described a good delineation of smaller vessels and used those data to estimate brain shift (60).

Our study was partially limited by the quality of the 3D iUS Doppler datasets, hampering the identification of landmarks. Additionally, we were confronted with several problems when segmenting the vascular tree. Preoperative MRI segmentation of the vessels was performed automatically in TOF or T1 contrast-enhanced sequences, or, alternatively, using the computer-assisted segmentation *via* the smart brush tool, depending on the quality of the auto-segmented objects. However, in 3D iUS Doppler ultrasound, neither automatic segmentation nor generation of objects using simple thresholding is currently available in the navigational software. Therefore, the vascular tree had to be manually segmented with the smart brush tool, which turned out to be a challenging and time-consuming procedure due to artifacts in the reconstructed and reformatted datasets. Whilst a rough segmentation and identification of vascular landmarks could be performed during surgery by either a neurosurgeon or a trained computer scientist experienced in this field within 5–10 minutes, thorough segmentation using the smart brush tool was more complex and not feasible during the surgical procedure at this stage. Segmentation of small perivascular vessels in ultrasound datasets, which could have been localized with power Doppler or CEUS, using the smart brush tool, and manual identification of corresponding landmarks (e.g., vascular bifurcation) in the peritumoral region would have prolonged the whole procedure dramatically, if even possible. Thus, we focused on greater vascular structures, which could be identified in color Doppler and did not include power Doppler or CEUS analysis. Nevertheless, given a future implementation of automatic ultrasound segmentation into the navigational software, both share a great potential in the field of local brain shift estimation by delineating small peritumoral vessels.

Another option would have been to utilize third-party applications for the analysis of the vessel tree and to transfer the data back to the navigational system. This would allow deeper exploration of approaches, as the one proposed by Reinertsen et al., who used vascular centerlines for the calculation of brain deformation (37, 46). However, here we focused on the built-in features of the navigational system to test for intraoperative applicability. To increase usability and efficiency, the analysis tools should be implemented into the navigational software itself, e.g., for automatic identification and correction of brain shift after 3D iUS Doppler acquisition.

This work involved only patients with temporal mass lesions. We chose this approach to allow good visualization of the vascular tree encompassing, if available, the internal carotid artery, the A1/A2 segment of the anterior cerebral artery, and the M1/M2 segment of the medial cerebral artery, and to make our result more comparable to each other. On the other hand, this patient inclusion criterion makes it difficult to generalize the study results to differently located tumors, in which visualization of the vascular tree may be less successful. Eventually, this study cannot describe the impact of 3D iUS Doppler on every kind of brain tumor resection, but only for those cases in which the deeply located vascular structures are easily depictable in Doppler imaging. Finally, due to the limited number of identified corresponding anatomical landmarks, the here described approach does not allow full correction of brain shift within the navigational system yet. Identification of more representative landmarks or vascular segments would allow fusion of preoperative and intraoperative imaging as shown by Rasmussen et al., who have found satisfactory results in five cases of automatic multimodal fusion of preMRI and i3D US Doppler postoperatively and proposed intraoperative use of their technique (45).

Despite these limitations, we found 3D iUS Doppler to be a valuable adjunct to our multimodal navigational setup. In the current setup, identification of vascular landmarks in color Doppler imaging allows intraoperatively estimation of local brain shift and thus increases safety during resection. Future implementation of automatic segmentation in ultrasound imaging or the possibility of generating objects *via* simple thresholding would facilitate further analysis of the vascular anatomy and brain shift and might allow navigational updating using 3D iUS Doppler datasets. Under these conditions, the advantages of power Doppler mode and CEUS could be fully exploited, and it will be of great interest to find out which type of vascular imaging contributes best and most feasibly to the determination of brain shift.

## Data Availability Statement

The original contributions presented in the study are included in the article/supplementary material. Further inquiries can be directed to the corresponding author.

## Ethics Statement

The studies involving human participants were reviewed and approved by Ethikkommission der Philipps Universität Marburg, Fachbereich Medizin. The patients/participants provided their written informed consent to participate in this study.

## Author Contributionsn

Conceptualization, MB, CN, BS. Methodology, MB and BS. Validation, BC, CN, and MP. Investigation, MB, BS, and DZ. Resources, MB, BC, CN, MP, BS, and DZ. Data curation, MB and BS. Writing—original draft preparation, BS. Writing—review and editing, MB and CN. Visualization, MB and BS. Supervision, MB, CN, and BS. Project administration, MB. All authors contributed to the article and approved the submitted version.

## Conflict of Interest

CN and MB are consultants for Brainlab.

The remaining authors declare that the research was conducted in the absence of any commercial or financial relationships that could be construed as a potential conflict of interest.

## Publisher’s Note

All claims expressed in this article are solely those of the authors and do not necessarily represent those of their affiliated organizations, or those of the publisher, the editors and the reviewers. Any product that may be evaluated in this article, or claim that may be made by its manufacturer, is not guaranteed or endorsed by the publisher.
